# Paradoxical Hyperexcitability in Disorders of Neurodevelopment

**DOI:** 10.3389/fnmol.2022.826679

**Published:** 2022-04-29

**Authors:** Michelle W. Antoine

**Affiliations:** Section on Neural Circuits, National Institute on Alcohol Abuse and Alcoholism, National Institutes of Health, Bethesda, MD, United States

**Keywords:** seizures, autism, excitation, Rett, Angelman

## Abstract

Autism Spectrum Disorder (ASD), Rett syndrome (RTT) and Angelman Syndrome (AS) are neurodevelopmental disorders (NDDs) that share several clinical characteristics, including displays of repetitive movements, developmental delays, language deficits, intellectual disability, and increased susceptibility to epilepsy. While several reviews address the biological basis of non-seizure-related ASD phenotypes, here, I highlight some shared biological mechanisms that may contribute to increased seizure susceptibility. I focus on genetic studies identifying the anatomical origin of the seizure phenotype in loss-of-function, monogenic, mouse models of these NDDs, combined with insights gained from complementary studies quantifying levels of synaptic excitation and inhibition. Epilepsy is characterized by a sudden, abnormal increase in synchronous activity within neuronal networks, that is posited to arise from excess excitation, largely driven by reduced synaptic inhibition. Primarily for this reason, elevated network excitability is proposed to underlie the causal basis for the ASD, RTT, and AS phenotypes. Although, mouse models of these disorders replicate aspects of the human condition, i.e., hyperexcitability discharges or seizures on cortical electroencephalograms, measures at the synaptic level often reveal deficits in excitatory synaptic transmission, rather than too much excitation. Resolving this apparent paradox has direct implications regarding expected outcomes of manipulating GABAergic tone. In particular, in NDDs associated with seizures, cortical circuits can display reduced, rather than normal or increased levels of synaptic excitation, and therefore suggested treatments aimed at increasing inhibition could further promote hypoactivity instead of normality. In this review, I highlight shared mechanisms across animal models for ASD, RTT, and AS with reduced synaptic excitation that nevertheless promote hyperexcitability in cortical circuits.

## Introduction

Rett syndrome (RTT) is an X-linked disorder that in ∼95% of cases is caused by mutations in the gene encoding the transcription factor Methyl-CpG-binding protein 2 (*MECP2*) (Amir et al., 1999). RTT occurs almost exclusively in females and is characterized by largely normal development for the initial 6-18 months after birth, followed by regression of previously acquired speech and motor skills (Guy et al., 2001). Epileptic seizures of various types (for example: tonic-clonic, absence, myoclonic, tonic) occur in 60-80% of Rett syndrome patients (Operto et al., 2019). Typically, seizure onset initiates between 2 and 3 years of age, with seizure severity often increasing until 7-12 years of age (Glaze et al., 2010).

Like Rett Syndrome, Angelman Syndrome (AS) is a rare neurodevelopmental disorder that affects ∼1/10,000 to 1/20,000 individuals, and is characterized by developmental delay, intellectual disability, increased susceptibility to seizures, impaired language development, and movement or balance dysfunction (Kishino et al., 1997; Matsuura et al., 1997; Williams, 2005). Approximately, 5-15% of AS cases are attributed to reduced or absent maternal expression on chromosome 15q of the gene *UBE3A*, which encodes the E6-AP ubiquitin ligase, an enzyme that links ubiquitin to specific proteins thereby targeting them for degradation within the cell proteasome (Huibregtse et al., 1993; Horsthemke and Wagstaff, 2008). These pathogenic point mutations in UBE3A are more common in familial rather than sporadic patients and though several seizure types occur, atypical absence seizures and myoclonic seizures are the more common forms.

Autism spectrum disorder shares aspects with both RTT and AS. Of the three disorders, ASD is the most common, currently affecting 1 in 54 eight-year-old children in the United States (Knopf, 2020). Unlike, RTT and AS, hundreds of genes have been identified that increase susceptibility to developing ASD. Of these genes, loss-of-function mutations in *SCN2A*, which encodes the Na_v_1.2 voltage-gated sodium channel pore-forming alpha II subunit, has one of the strongest genetic associations with ASD susceptibility (Satterstrom et al., 2020). Functionally, Na_v_1.2 is involved in the initiation and propagation of action potentials in immature neurons throughout the central nervous system (Catterall, 2017) and appears essential for spiking in a subpopulation of immature oligodendrocytes (Gould and Kim, 2021). The development of patients with SCN2A mutations and ASD is mostly typical until 6-months of age, after which motor and verbal delays manifest, and between 18 months to 4 years of age, one-third of these cases will display seizures or milder and/or later-onset epilepsies (Wolff et al., 2017; Sanders et al., 2018). Here, we focus on mechanisms that may help drive these milder and/or later-onset epilepsies.

Loss-of-function mutations in *MECP2*, *UBE3A*, and *SCN2A* yield a shared neurodevelopmental disorder phenotype that strongly features abnormal electroencephalograms (EEGs) and increased susceptibility to seizures. It is often theorized that this increased association with epilepsy reflects a shared biological mechanism. Typically, this mechanism is proposed to involve excess synaptic excitation that is largely driven by too little inhibition at the synaptic and circuit levels (Rubenstein and Merzenich, 2003). To date, several molecular and physiology-based studies in mouse models carrying loss-of-function mutations in either *Mecp2*, *Ube3A* or *Scn2a* have demonstrated key aspects of the human phenotype, i.e. the occurrence of either hyperexcitability discharges or seizure activity on cortical electroencephalograms (Born et al., 2017; Ogiwara et al., 2018; Wither et al., 2018). Unexpectedly, measures at the synaptic level also revealed a shared phenotype for deficient, rather than excess, excitatory synaptic transmission (Dani et al., 2005; Wood and Shepherd, 2010; Wallace et al., 2012; Banerjee et al., 2016; Spratt et al., 2019). In the ensuing sections of this review, guided first by Cre/Lox genetic studies that identify the causal cell source(s) of the seizure or hyperexcitability phenotype, and second by parallel studies in these mice that quantify synaptic excitation and inhibition levels, I propose that shared mechanisms account for hyperexcitability discharges or seizure activity in ASD, RTT and AS despite impaired excitatory synaptic transmission.

### A Shared Cortical Locus for Absence Seizure Susceptibility in *Mecp2* and *Scn2a* Haploinsufficiency

In the human condition, a key diagnostic criterion for absence epilepsy is the display of spike-and-wave discharges (SWDs) on cortical electroencephalograms (EEGs) (Terlau et al., 2020). SWDs are defined by a characteristic electroencephalographic pattern consisting of a sharp, negative spike followed by a slow wave. Unlike other seizure phenotypes, absence seizures typically lack overt displays of convulsions. Instead, during seizures, EEGs may indicate bilaterally synchronized 3–4 Hz SWDs while electromyography (EMG) measures show brief periods of behavior arrest (Panayiotopoulos, 2001; Blumenfeld, 2005; Idro et al., 2008). Mice with a heterozygous deletion of *Scn2a* (*Scn2a^+/–^*) recapitulate the human phenotype of “absence-like” seizures (Ogiwara et al., 2018). Electrocorticogram (ECoG)-EMG recordings in *Scn2a^+/–^* mice at 10-27 weeks of age reveal occurrences of synchronous, bilateral, cortical SWDs during behavioral quiescence. As *Scn2a* haploinsufficiency reduces the Na_v_1.2 current by half, the projected result is reduced neuronal excitability, but surprisingly seizure activity is observed.

Dissection of the cell types underlying this seizure phenotype with Cre-lox genetics revealed critical contributions of Na_v_1.2 haploinsufficiency in neurons that are excitatory rather than inhibitory. In this experiment, *Emx1*^*Cre*/+^;*Scn2a* ^*fx*/+^ mice were generated from a cross between *Emx1*-Cre mice (Gorski et al., 2002) and the *Scn2a* floxed allele (Ogiwara et al., 2018), and showed reduced expression of *Scn2a* in dorsal-telencephalic derivatives (e.g., neocortical and hippocampal excitatory neurons). ECoG-EMG recordings in 6–8 week-old *Emx1*^*Cre*/+^;*Scn2a* ^*fx*/+^ mice showed “absence-like” seizures featuring SWDs with EMG suppression. Like the human condition, administration of the anticonvulsant ethosuximide suppressed seizures (Ogiwara et al., 2018). In contrast, this “absence-like” seizure phenotype was not observed when using the vesicular GABA transporter Cre mouse line (Vgat-Cre) to reduce Na_v_1.2 expression specifically in interneurons (Ogiwara et al., 2018; Miyamoto et al., 2019). Thus, a loss of *Scn2a* from GABAergic cell types may not be a primary cause underlying SWDs.

Finer resolution cell-type dissection of the seizure phenotype showed that SWDs can be generated in mice with a reduction of *Scn2a* in cortical layer (L)5 pyramidal neurons (i.e., *Trpc4*^*Cre*/+^;*Scn2a* ^*fx/fx*^ mice) but not in *Ntsr*^*Cre*/+^;*Scn2a* ^*fx/fx*^ mice, where *Scn2a* expression is reduced in cortical L6 neurons (Miyamoto et al., 2019). These findings in mice support studies conducted in rat models of absence epilepsy, which suggest that the deep layers of the somatosensory cortex may act as the initiation site of SWDs (Blumenfeld, 2005; Pinault and O’Brien, 2005; Lüttjohann and van Luijtelaar, 2015; Depaulis and Charpier, 2018; McCafferty et al., 2018). Thus, the pathological origins of “absence-like” seizures in rats may be similar to the mouse ASD condition.

Whole-cell current clamp recordings in adult L5b PYR neurons deficient in *Scn2a*, due to AAV-EF1a-Cre-mCherry virus-mediated recombination of the floxed allele (Scn2a*^fx/fx^*), indicated that cell excitability was elevated. Namely, the number of action potentials increased in response to somatic depolarizing current injections, input resistance increased, action potential (AP) repolarization speed decreased, and the afterhyperpolarization (AHP) during spike trains was more depolarized, when compared to wild type (Scn2a^*fx*/+^ or Scn2a^*fx/fx*^) neurons in the contralateral uninfected hemisphere of the medial prefrontal cortex (mPFC) (Spratt et al., 2021). Compartmental modeling revealed two interesting findings. The first and main finding was that complete deletion of Na_v_1.2 promoted an exponential rather than linear shift in the relationship between the Na_v_ and K-channel current during an AP (Spratt et al., 2021). This effect can potentially increase overall L5 excitability because repolarization in neurons is incomplete between APs, so threshold attainment and AP generation can occur more rapidly. The second finding was that increased input resistance can shift the onset of the Firing/Current (F/I) curve left, which can theoretically increase the number of APs generated at current onset. Intriguingly, during this current onset period, *Scn2a* deficient neurons regularly produce high-frequency bursts (Spratt et al., 2021).

Besides L5b PYR excitatory neurons, *Scn2a* is expressed in somatostatin-positive (SSt^+^) and vasoactive intestinal peptide-positive (VIP^+^) interneurons, as well as in unmyelinated axons of GABAergic medium spiny neurons (MSNs) (Li et al., 2014; Miyazaki et al., 2014; Yamagata et al., 2017). It is currently unclear whether excitability is similarly altered in SSt^+^ and VIP^+^ interneurons under conditions where *Scn2a* is severely reduced or absent. However, another recent study, using a mixed striatal MSN population, linked drastically reduced *Scn2a* levels to an increase in intrinsic excitability. Patch-clamp recordings in brain slices from an Na_v_1.2 hypomorph mouse (Scn2a*^gt/gt^*) (Eaton et al., 2021) with 25-30% of the wild-type protein level, revealed enhanced neuronal excitability in striatal MSNs (i.e., an increased number of action potentials/spikes generated in response to depolarizing current injections, reduced rheobase, a mildly elevated fast after-hyperpolarization, and increased input resistance) (Zhang et al., 2021). *Ex vivo* recordings in adult MSNs, with sparse rescue of the severe Nav1.2 deficiency via viral transduction, showed that these alterations to intrinsic excitability were largely cell autonomous and likely driven by the downstream effects of a reduction in the total potassium current (Zhang et al., 2021). Spratt et al. (2021) did not observe changes in potassium channel function in L5 cortical neurons. Nevertheless, both studies identified largely cell autonomous changes in input resistance and AHP hyperpolarization that may contribute to epilepsy in cases of severe *Scn2a* deficiency, even though seizure activity was not reported specifically for Scn2a*^gt/gt^* mice (Eaton et al., 2021; Zhang et al., 2021). Studies on human neurons also show evidence of paradoxical hyperexcitability. Induced pluripotent stem cell derived *SCN2A^–/–^* neurons when compared with isogenic controls display an increased number of spikes on multielectrode arrays and impaired excitatory synapse formation and function (Brown et al., 2021). Key observations from mouse studies were also replicated with human neurons, including increased input resistance, reduced AP height, and reduction in the somatic component of the rising phase of the AP. However, unlike in mice, only a minor increase in the number of APs generated at current onset was observed (Brown et al., 2021). Excitatory neurons derived from an ASD patient heterozygous for the R607* truncating mutation in *SCN2A* also showed reduced excitatory synapse function. Future studies incorporating measures of synaptically-evoked spiking and excitatory currents (which are reduced in *Scn2a* haploinsufficiency and presumably in more severe *Scn2a* deficiencies), will help to confirm the predicted effects of physiological changes on seizure-related behaviors.

Similar to *Scn2a^+/–^* mice, adult female mice heterozygous for a null mutation in *Mecp2* (*Mecp*2^+/^*^–^*) exhibit the cardinal hallmarks of “absence-like” seizures, i.e., the occurrence of brief (≤ 2 s) spontaneous cortical epileptiform discharges with frequencies of 6-8 Hz in the somatosensory cortex during acute behavioral arrest with sensitivity to ethosuximide (Wither et al., 2018). Here too, Cre-lox genetics was used to dissect potential causal roles of *Mecp2* loss in excitatory and GABAergic neurons in the seizure phenotype. Mice in which calcium-calmodulin-dependent protein kinase II (CaMKII)-Cre (Chen et al., 2001) was used to delete *Mecp2* (Chen et al., 2001) selectively in forebrain excitatory neurons during early postnatal development reproduce RTT associated features such as increased anxiety, impaired motor coordination, and social deficits (Gemelli et al., 2006). However, the effects on seizures were not reported.

Like the *Scn2a* mutant condition, deleting the floxed *Mecp2* allele from excitatory neurons and glia in the neocortex and hippocampus using the *Emx1-Cre* mice (*Emx1*^*Cre*/+^; *Mecp2* ^*fx/y*^) results in detection of absence seizures during EEG recordings between 6-8 weeks of age (Zhang et al., 2014). Although, the authors attributed the phenotype to *Mecp2* reduction in neurons rather than glia, intriguingly, *Mecp2* is expressed in astrocytes throughout the brain. Indeed, it has been proposed that while neurons may initiate several symptoms of RETT syndrome, loss of *Mecp2* in astrocytes may strongly affect disease progression (Lioy et al., 2011). Loss of MECP2 from astrocytes, using the floxed *Mecp2* allele (Chen et al., 2001) and the *hGFAPcreT2* mouse line (Hirrlinger et al., 2006) results in a smaller body size, clasped hindlimb posture and irregular breathing (Lioy et al., 2011). Notably, as the *hGFAPcreT2* mouse line does not recombine very well in the cortex and the substantia nigra, this phenotype could be potentially more severe upon additional loss of *Mecp2* expression from these brain regions (Lioy et al., 2011). However, the authors buttress their premise that altered neuronal physiology mediated the “absence-like” seizure phenotype by showing that although the intrinsic excitability, resting potential, input resistance and spike threshold of L5 pyramidal neurons in the cortex is normal, mild depolarizations in the membrane potential (i.e., from −59 mV to −53 mV) produced significantly more spikes in the *Emx1*^*Cre*/+^;*Mecp2* ^*fx/y*^ mice than in controls due to reduced inhibitory tone (Zhang et al., 2014). As we will discuss in the next section of the review, this reduction in inhibitory tone may arise from the combined effects of reductions in inhibitory inputs onto postsynaptic excitatory PYR neurons and cell autonomous changes in astrocytes that diminish synaptic GABA levels. Nevertheless, hyperactivity of cortical L5 pyramidal neurons may be a central mechanism that drives seizures when either *Scn2a* or *Mecp2* gene expression is absent or reduced.

### Dual Roles for Reduced Inhibition in Seizure Susceptibility and Spiking Stability

Absence seizures in *Scn2a* or *Mecp2* cortical mouse models have been linked to hyperactivity of cortical L5 pyramidal (PYR) neurons (Zhang et al., 2014; Ogiwara et al., 2018; Miyamoto et al., 2019). However, the mechanism underlying hyperexcitation warrants further dissection. Studies on *Emx1*^*Cre*/+^;*Mecp2* ^*fx/y*^ mice proposed a model whereby MECP2 loss in cortical PYR neurons causes a reduction of GABAergic transmission, driving hyperactivity (Zhang et al., 2014). Whole-cell patch-clamp recording in L5 PYR neurons in both the mPFC and somatosensory cortex of acute brain slices from *Emx1*^*Cre*/+^;*Mecp2* ^*fx/y*^ mice at P17–P20 showed that although the amplitude and the kinetics of spontaneous IPSCs (sIPSCs) and miniature(m) IPSCs were unchanged, the frequency of both parameters were reduced (Zhang et al., 2014). In mutant neurons, measures of evoked monosynaptic IPSCs (i.e., recorded in the presence of DNQX and kynurenic acid) at P18-P20 showed stable IPSC thresholds but significantly reduced peak IPSC amplitudes when compared to control neurons. Notably, this reduction in inhibition is long-lasting and remained as mice aged to 6–7 weeks (Zhang et al., 2014). Comparable changes in immunofluorescent staining of puncta for the vesicular GABA transporter (VGAT, a marker for GABAergic synaptic terminals) in L5 PYR neurons from *Emx1*^*Cre*/+^;*Mecp2* ^*fx/y*^ mice were also observed, with a 42% reduction in puncta at P18-P19 and a 37% reduction at 7–8 weeks of age. In L2/3 PYR somatosensory cortical neurons, sIPSC frequency reduced by 57% in mutant neurons, and sIPSC amplitude decreased by 20%. Interestingly, these observed reductions in synaptic inhibitory currents were not replicated when *Mecp2* was deleted from forebrain GABAergic neurons using the *Dlx5/6-Cre* mice (Zhang et al., 2014). Overall, these results show that the loss of *Mecp2* in PYR cortical neurons causes a reduction in GABAergic transmission onto postsynaptic excitatory neurons.

**FIGURE 1:**
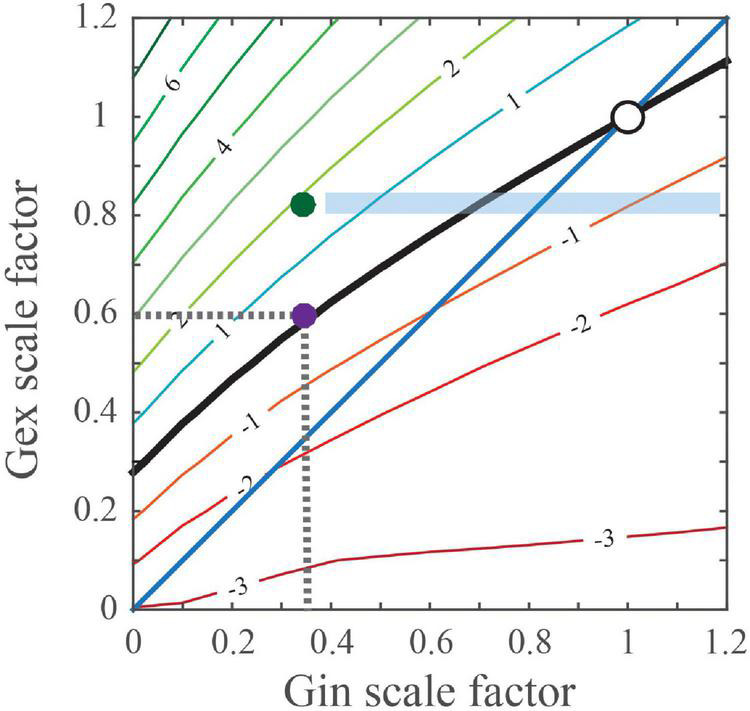
Predicted changes in excitatory and inhibitory conductances that maintain a normal/wild type-sized PSP. Simulation calculates the effect of differentially scaled excitatory (Gex) and inhibitory (Gin) conductance combinations on the peak change in the membrane potential (Vm) of postsynaptic cortical PYR neurons. Points on the black ‘PSP stability contour’ line comprise the postsynaptic Gex and Gin conductance combinations that maintain a wild type-sized PSP. For example, the purple dot (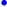) on the contour line indicates that inhibition was reduced by 65%, which is indicated as 0.35 on the x-axis, whereas excitation was reduced by 40% and is indicated as 0.6 on the y-axis. The blue diagonal or unity line indicates numerically equal changes in Gex and Gin conductances. For regions where the contour rests above the diagonal, when Gex decreases, Gin must decrease more than Gex to maintain a normal-sized PSP. Compensatory changes in Gin can potentially be imperfect, maintaining balance in one brain region but not another. The green dot (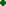) illustrates a hypothetical case where Gex is reduced but features of hyperexcitability are present, i.e., PSPs are 2 mVs larger. For this case, the blue shaded rectangular area predicts that pharmacological treatments selectively increase synaptic GABA, may induce circuit hypoexcitability as Gin levels near normal.

Remarkably, an identical phenotype of a reduction in GABAergic transmission onto postsynaptic CA1 excitatory PYR neurons was observed after PYR-specific deletion of the ASD-related gene, tuberous sclerosis complex 1 (*Tsc1*) (Bateup et al., 2013). The authors also suggested that such reductions in inhibition could alone drive Tsc1-related hyperexcitability at the network level to result in seizures. However, an alternative explanation exists. Namely, that these changes in inhibition are compensatory, rather than being a primary defect that directly drives seizure activity. It was previously shown that a primary deficit in either excitatory synaptic input or intrinsic excitability in excitatory neurons can induce a decrease in inhibitory inputs onto these neurons to balance the levels of synaptic excitation and inhibition (Xue et al., 2014; He et al., 2018). In this activity-dependent phenomenon, decreases in synaptic excitation are matched by greater reductions in synaptic inhibition levels to preserve the magnitude of cell depolarization and maintain spiking stability (Antoine et al., 2019). Figure 1, adapted from Antoine et al. (2019), illustrates results of a simulation examining the effect of differently scaled excitatory (Gex) and inhibitory (Gin) conductance combinations on the peak change in the membrane potential (Vm) in postsynaptic cortical PYR neurons. Indicated in Figure 1 is the black “PSP stability contour” line illustrating the postsynaptic Gex and Gin conductance combinations that maintain a wild type-sized PSP peak. Notably, Gin changes primarily reflect the alterations to parvalbumin- positive (PV^+^) interneuron mediated inhibition onto postsynaptic cortical PYR neurons. In regions where the contour rests above the blue diagonal or unity line, when Gex decreases, Gin must decrease more than Gex to maintain a normal PSP peak.

Thus, it is possible that the observed reduction in GABAergic transmission onto postsynaptic excitatory neurons in *Tsc1* and/or *Mecp1* reflects a change that compensates for either diminished excitatory drive or intrinsic excitability in PYR neurons, rather than to directly drive seizure activity. In line with this alternative interpretation, current clamp recordings from *Tsc1*-deficient CA1 PYR excitatory neurons revealed that mutant neurons are significantly less excitable than control neurons: exhibiting reduced action potential firing in response to a wide-range of depolarizing current injections, increased action potential threshold, decreased membrane resistance and increased capacitance (Bateup et al., 2013). Furthermore, in response to increasing stimulus intensities, no significant difference in either the amplitude of excitatory postsynaptic potentials (EPSPs) or firing frequencies were observed. Thus, these cell autonomous reductions in GABAergic transmission onto postsynaptic excitatory neurons may not be sufficient to drive circuit hyperexcitability when they co-occur with either diminished excitatory input or reduced intrinsic excitability in PYR neurons. In line with this conclusion, *Tsc1* mutations that promote increases in PYR intrinsic excitability co-occurs with hyperexcitability (Abs et al., 2013; Koene et al., 2021).

Multiple studies reveal a cell autonomous reduction in excitatory drive or activity in cortical and hippocampal PYR neurons with *Mecp2* deficiency (Chao et al., 2007; Blackman et al., 2012; Qiu et al., 2012; Meng et al., 2016). To determine the functional role of *Mecp2* in PYR glutamatergic neurons, Meng and colleagues used the vesicular glutamate transporter 2-Cre mice (Vglut2^*Cre*/+^, Vong et al., 2011) to delete the conditional *Mecp2* allele (Guy et al., 2001) from excitatory neurons throughout the brain. Whole-cell patch-clamp recordings from cortical L5 PYR neurons in the somatosensory cortex of mutant 6- to 8-week-old mice showed reduced spontaneous action potential firing (Meng et al., 2016). Measurements of postsynaptic currents (PSCs) showed a drop in both spontaneous excitatory postsynaptic currents (sEPSCs) and miniature excitatory postsynaptic currents (mEPSCs) compared to controls. Consistent with the idea that PYR neurons cell autonomously decrease inhibitory inputs to compensate for reduced levels of excitation, reductions in spontaneous inhibitory postsynaptic currents (sIPSCs) were also observed. In an otherwise *Mecp2* null mouse containing no MECP2 protein throughout the brain and body, restoration of *Mecp2* only in Vglut2*^Cre^* excitatory neurons normalized spontaneous action potential firing and mEPSCs to control levels (Meng et al., 2016).

C57BL/6J mice that are heterozygous for *Ube3a*, where the deficient allele is inherited by the mother (m) rather than the father (p) (*Ube*3*a^m^*^–/p+^), serve as a mouse model for Angelman Syndrome (AS) (Jiang et al., 1998; Wallace et al., 2012). Electrophysiology studies on these mice by Wallace and colleagues, provided evidence that preferential decreases in inhibitory inputs that co-occur with decreases in excitatory input or PYR activity may not be sufficient to induce a seizure phenotype. Whole-cell patch-clamp recordings from neocortical slices in L2/3 pyramidal (PYR) neurons of the visual cortex in WT and *Ube*3*a^m^*^–/p+^ mice at postnatal day (P) 80, showed no genotypic difference in the amplitude of either miniature (m)EPSCs or mIPSCs (Wallace et al., 2012). However, decreases in the frequency of both mEPSCs and mIPSCs were observed, with a preferential 50% reduction in spontaneous inhibitory synaptic activity and a 28% decrease in excitatory synaptic activity (Wallace et al., 2012). Interestingly, when mIPSC recordings are performed at an earlier age during the critical period for ocular dominance plasticity, both the amplitude and the frequency of mIPSCs in these P21–P28 mice appear normal. Consistent with inhibitory changes serving a compensatory role, excitatory deficits are observed during the critical period prior to observed changes in inhibition. Furthermore, synaptic deficits may be limited to the excitatory neurons, as neither the amplitude nor the frequency of miniature EPSCs or mIPSCs onto fast-spiking inhibitory interneurons was affected. Analogous to the data of the mIPSC recordings, reductions in L4-evoked inhibitory postsynaptic currents on L2/3 PYR neurons (eIPSCs) were also observed at P80 but not at P25 (Wallace et al., 2012). Despite decreases in both excitatory and inhibitory synaptic activity, spontaneous network activity and baseline firing rates were not altered by the loss of *Ube3a* expression. Likewise, recordings of spontaneous local network activity in L2/3 regular spiking (RS) putative pyramidal neurons of the visual cortex during presentation of a gray screen, showed that neither average spontaneous firing rates nor UP state frequency and duration were significantly different between wild type and mutant mice (Wallace et al., 2017).

Overall, these results show that the loss of *Mecp2*, *Tsc1*, and *Ube3a* in postsynaptic excitatory cortical neurons can induce preferential reductions in inhibition that may have a compensatory role that is aimed at stabilizing synaptic activity, rather than the deficiencies in inhibition directly driving seizures. Thus, these alterations in inhibition alone may not be sufficient to cause seizures. What additional factors might be required? Despite employing Cre-lox approaches to delete *Mecp2* from glutamatergic excitatory neurons in both *Emx1*^*Cre*/+^;*Mecp2*^*fx/y*^ and Vglut2^*Cre*/+^;*Mecp2*^*fx/y*^ mutants, both mice exhibit different levels of seizure severity. Video-EEG recordings at 10-weeks of age showed that ∼37.5% of Vglut2^*Cre*/+^;*Mecp2*^*fx/y*^ mice displayed SWDs at a frequency of 3.3 ± 2.1 episodes/hour and with an episode duration of 4.1 ± 0.4 s (Meng et al., 2016). In contrast 100% of *Emx1*^*Cre*/+^;*Mecp2*^*fx/y*^ mutant mice, displayed spike-and-wave discharges at a frequency of 36 ± 7 episodes/hour and episode duration of 1.3 ± 0.1 s (Zhang et al., 2014).

At least two differences may account for the variance in seizure severity. Firstly, in addition to glutamatergic excitatory neurons, *Emx1*^*Cre*/+^ deletes *Mecp2* in astrocytes. In fact, cell-specific deletion of *Mecp2* in astrocytes significantly reduces ambient GABA levels in the extracellular space and decreases tonic inhibition through increases in GAT3 GABA transporter expression (Dong et al., 2020).

Therefore, the loss of *Mecp2* in astrocytes could exacerbate reductions in synaptic inhibition beyond the point needed to compensate for the initial decline in synaptic excitation. In support of non-glutamatergic *Mecp2*-deficient cell types contributing to seizure phenotypes in *Mecp2* null mice, restoration of *Mecp2* in Vglut2^Cre^ excitatory neurons only slightly reduces seizure incidence by ∼12.5%.

Secondly, the *Emx1^Cre^* and the Vglut2*^Cre^* drivers recombine in some non-overlapping brain regions at different developmental timepoints and at different recombination efficiencies. For example, unlike *Vglut2*^*Cre*/+^;*Mecp2* ^*fx/y*^ mice, *Mecp2* is not deleted in the thalamus or cerebellum of *Emx1*^*Cre*/+^;*Mecp2* ^*fx/y*^ mice. Therefore, it is possible that the loss of *Mecp2* from these additional brain areas in the *Vglut2*^*Cre*/+^;*Mecp2* ^*fx/y*^ mice contributed to effects that help mitigate circuit hyperexcitability. Alternatively, *Emx1*^*Cre*^ recombines in cells at early stages of the cortical PYR cell lineage, whereas *Vglut2*^*Cre*^ recombines in essentially mature PYR neurons. Thus, it is possible that *Mecp2* plays a role in PYR neuron maturation as well as activity. Regardless of the genetic explanations for the differing severities in the *Emx1*^*Cre*^ and *Vglut2*^*Cre*^ driven phenotypes, differences in the extent to which PYR excitatory drive or activity is reduced may have contributed to the pointedly increased seizure severity in the *Emx1*^*Cre*/+^;*Mecp2* ^*fx/y*^ mutant mice. In the next section, we will explore the effects of ASD gene deletion in inhibitory neurons on circuit hyperexcitability and seizure susceptibility.

### Contributions of Reductions in Inhibition Due to the Dysfunction of Inhibitory Neurons to Circuit Hyperexcitability and Seizure Susceptibility

Two independent studies showed that seizures or epileptiform activity are not produced in mice where a forebrain-specific GABAergic Cre (i.e., *Dlx5/6-Cre*; Monory et al., 2006) was used to delete *Mecp2* from inhibitory neurons (Chao et al., 2010; Zhang et al., 2014). The *Dlx5* and *Dlx6* promoter is expected to drive Cre expression in GABAergic interneurons (including parvalbumin-positive (PV^+^), somatostatin-positive (SSt^+^+), and vasoactive intestinal peptide-positive (VIP^+^) interneurons) throughout the cerebral cortex, striatum, and hypothalamus (De Lombares et al., 2019). However, additional Cre-lox studies to elucidate the cause of the seizures showed that the loss of *Mecp2* from SSt^+^ interneurons throughout the brain via SSt^*Cre*/+^ mice (Taniguchi et al., 2011) resulted in spontaneous seizures but with a substantially delayed onset (Ito-Ishida et al., 2015). Hence, *Mecp2* loss from interneurons that are not located in the forebrain may be needed exclusively, or perhaps required in combination with *Mecp2* loss from forebrain interneurons to drive hyperexcitability or the seizure phenotype. At 15-weeks of age, almost 10% of *SSt*^*Cre*/+^;*Mecp2*^–/*y*^ mice developed spontaneous epileptic seizures and by 45-weeks of age 50% percent of these mice developed spontaneous epileptic seizures (Ito-Ishida et al., 2015). Generalized tonic clonic seizures were also observed in *SSt*^*Cre*/+^;*Mecp2*^–/*y*^ mice during routine handling (Ito-Ishida et al., 2015). As these seizure phenotypes were not observed upon deletion of *Mecp2* from forebrain SSt^+^ interneurons alone, these results suggest that SSt^+^ neurons in the midbrain and/or hindbrain may play a particularly salient role in seizure generation. In contrast, the loss of *Mecp2* from parvalbumin positive (PV^+^) interneurons, using a Pvalb-2A-Cre driver (Madisen et al., 2010), did not produce seizures (Ito-Ishida et al., 2015). The lack of a phenotype was highly unexpected, especially given that fast-spiking PV^+^ neurons provide a major source of inhibition that regulates pyramidal cell output (Atallah et al., 2012; Kepecs and Fishell, 2014). More importantly, PV^+^ neurons, rather than SSt^+^ neurons, play a dominant role in maintaining the excitatory/inhibitory balance in the cortex (Xue et al., 2014; Ito-Ishida et al., 2015).

More widespread deletion of *Mecp2* from all GABAergic and glycinergic neurons, using the Viaat-Cre mouse line, further outlines a subtler role for the cell autonomous loss of *Mecp2* from interneurons in driving seizures. Interestingly, deletion of *Mecp2* expression using Viaat-Cre produces frequent hyperexcitability discharges but no seizures (Chao et al., 2010). Immunohistochemical and electrophysiological analyses reveal subtle disruptions of GABAergic neuronal function, namely reduced mRNA transcript levels for the genes encoding enzymes involved in the conversion of glutamate to GABA (*Gad1* and *Gad2*), reduced GABA immunoreactivity, and reduced mIPSCs amplitude and charge (Chao et al., 2010). *Mecp2* re-expression in Viaat-Cre expressing neurons in the *Mecp2* null normalized mRNA levels of Gad1 and Gad2 but the reduction in mEPSC amplitude remained (Ure et al., 2016). Furthermore, the incidence of non-seizure hyperexcitability discharges and seizures were dramatically reduced (Ure et al., 2016). Collectively, these data suggest that loss of *Mecp2* from multiple cell types (i.e., glutamatergic neurons, astrocytes, and interneurons) reduce inhibition levels to a range that may drive seizures at early postnatal developmental stages, but restoration of *Mecp2* in inhibitory neurons may be sufficient to elevate inhibition above the seizure induction threshold.

A recent contrasting report is worth discussing. This study, in which the same floxed *Mecp2* allele (Guy et al., 2001) as above was purportedly recombined with Cre lines targeting interneurons, namely *Dlx5/6-Cre* mice (Monory et al., 2006), SSt*^Cre^* mice (Taniguchi et al., 2011), *Pvalb^Cre^* mice (Hippenmeyer et al., 2005) and *Vip^Cre^* mice (Taniguchi et al., 2011), detected seizure activity in each conditional *Mecp2* mutant line (Mossner et al., 2020). Seizures manifest in 100% of *Mecp2*^–/*y*^, 100% of *Dlx5/6-Cre;Mecp2* ^*fx/y*^, 52.9% of SSt^*Cre*/+^;Mecp2^–/*y*^, 35.0% of PV^*Cre*/+^;Mecp2^–/*y*^, and 37.5% of *VIP*^*Cre*/+^;*Mecp2* ^*fx/y*^ mutants. Seizures were also detected in *Mecp2*^*fx/y*^ controls. However, as described above, previous studies failed to detect either hyperexcitability discharges or seizures in *Dlx5/6-Cre;Mecp2* ^*fx/y*^ mice even at 39-weeks of age (Ito-Ishida et al., 2015). It should be noted that Dlx5/6-Cre produces maternal germline recombination for several alleles (Luo et al., 2020), providing a possible explanation for 100% expressivity of seizures in not only the *Mecp2^–/y^* null, but also the *Dlx5/6-Cre; Mecp2 *^fx/y^** mice in Mossner et al. (2020). Contributions from strain background may also play a role in modifying the phenotype. Despite this potential caveat, this study demonstrated that single-unit recordings from putative regular-spiking cortical excitatory PYR neurons in awake mice, have 3 fold-larger firing rates in *Dlx5/6-Cre;Mecp2 *^fx/y^**, *VIP*^*Cre*/+^;*Mecp2*^*fx/y*^ and SSt^*Cre*/+^; Mecp2^–/*y*^ mice (Mossner et al., 2020).

Interestingly, studies on the *Ube*3*a*^*m*−/*p* +^ heterozygotes on the C57BL/6J strain background provide evidence that genetic alterations in interneurons that reduce their activity, can alter the intrinsic properties of excitatory PYR neurons to increase their excitability (Wallace et al., 2017). Continuous video EEG recording (24 hours a day for 5 days) for spontaneous seizure activity showed a significant increase in the amplitude and frequency of spontaneous polyspike activity in the somatosensory cortex (146 ± 54.64 events/day in *Ube*3*a*^*m*−/*p* +^ mice compared to 1.04 ± 0.20 events/day in control mice) (Born et al., 2017). However, similar to the Viaat-Cre; *Mecp2 *^fx/y^** mice, despite this increase in abnormal epileptiform activity, no spontaneous seizures were recorded in the *Ube*3*a*^*m*−/*p* +^ mice. Equally, seizures induced by exposure to a 140 dB aversive auditory stimulus were not observed and seizures induced via kainic acid administration bore similar latencies in the wildtype and *Ube*3*a*^*m*−/*p* +^ mutants (Born et al., 2017). *In vivo* whole cell recordings of L2/3 PYR neurons to dissect the hyperexcitability phenotype, showed that *Ube3a* mutant mice exhibited mildly increased spiking activity following current injection compared with control mice. This increase in the intrinsic excitability of L2/3 PYR neurons in the visual cortex was attributed to an increase in membrane resistance as no differences occurred between groups in resting membrane potential. *In vitro* experiments replicated the finding; showing a modest increase in intrinsic excitability of L2/3 PYR neurons in *Ube3a*^*m*–/*p*+^ mice compared to wildtype (Wallace et al., 2012).

To address whether the increased intrinsic excitability in L2/3 PYR neurons was cell autonomous or arose as a compensatory result to a reduction in inhibitory synaptic activity, Wallace and colleagues used the Gad2-Cre line (Taniguchi et al., 2011) to conditionally reinstate *Ube3a* expression in all GABAergic neurons of Ube3a^*STOP/p*+^ mice. This Cre-mediated gene re-expression is facilitated by the removal of a STOP cassette inserted between exons 3 and 4 of *Ube3a* (Silva-Santos et al., 2015). Reinstatement of *Ube3a* restored intrinsic excitability to control levels and thus illustrated that increased intrinsic excitability in PYR neurons can be induced solely from *Ube3a* loss in interneurons.

## Conclusion

**FIGURE 2:**
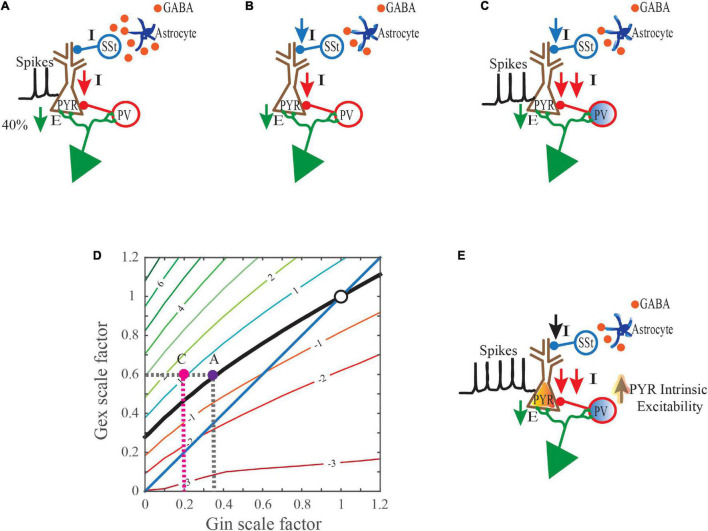
Collective mechanisms that may drive hyperexcitability when synaptic excitation levels are reduced. **(A)** A gene mutation that is linked to a neurodevelopmental disorder (NDD) induces cell autonomous changes in glutamatergic PYR neurons that reduce their excitatory postsynaptic input (E). This primary defect in the PYR neurons triggers cell autonomous decreases in inhibitory postsynaptic inputs (I). **(B)** The same NDD-linked gene mutation also induces cell autonomous changes in astrocytes that may reduce ambient GABA levels in the extracellular space. Reduced GABA levels may consequently lessen inhibitory output in select subsets of interneurons. In this example, somatostatin-positive (SSt^+^) neuron-mediated inhibition is reduced. **(C)** This NDD-linked gene mutation can also induce cell autonomous changes in parvalbumin-positive (PV+) interneurons which impair their inhibitory output. Moreover, these inefficiencies are perhaps exacerbated during incidences of strong or sustained inputs, as the ability of PV neurons to scale-up their responses is compromised. **(D)** In response to the 40% reduction in excitatory postsynaptic input from **(A)**, even less inhibition is now provided due to the combined effects of cell autonomous changes in astrocytes and PV+ interneurons. i.e. in C (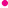 pink dot) an 80% drop, instead of the expected 65% that would typically occur in **(A)** (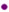 purple dot). The net effect is PSPs that are 1mV larger than normal and increased depolarization levels (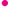 pink dot). **(E)** Events in **(D)** help drive an increase in the intrinsic excitability of PYR neurons.

Altogether, the studies discussed here implicate L5 of the visual, somatosensory, and prefrontal cortex as potential loci for further study into the mechanisms mediating seizure activity in ASD, RTT, and AS. Cortical L5 neurons integrate inputs from many sources (including L2/3 cortex) and output to both cortical and subcortical structures to mediate functions such as perception and movement. Future studies are required to further connect physiological abnormalities in L5 PYR neurons and their input and output sources to the generation of absence seizure-like activity (Miyamoto et al., 2019). While it is tempting to explain the cause of seizures solely in terms of deficits in inhibition, as illustrated in Figure 2, the overall source of the reduction can be quite complex. Some aspect of the total reduction in inhibition levels may compensate for reductions in either excitatory synaptic input or intrinsic excitability in glutamatergic PYR neurons. If left unchecked, circuits can become hypoactive. Decreases in inhibition that are mediated by cell autonomous effects in interneurons can prove more deleterious as they can enhance losses in inhibition and induce alterations to increase intrinsic excitability in glutamatergic PYR neurons. Other cell types such as astrocytes may also act to reduce inhibition levels. Studies of mice with seizure phenotypes where gene function is restored to one or more cell-types (e.g. interneurons, glia, excitatory PYR neurons) in an otherwise null background could significantly aid in our efforts to identify the most crucial cell-types for targeted therapies. Additional insights into the acute and long-term consequences of gene deficiencies on behavior and brain physiology are also needed to ensure that cell type targeted pharmacologic or genetic therapies do not disrupt compensatory mechanisms and worsen the severity of symptoms.

## Author Contributions

The author confirms being the sole contributor of this work and has approved it for publication.

## Conflict of Interest

The author declares that the research was conducted in the absence of any commercial or financial relationships that could be construed as a potential conflict of interest.

## Publisher’s Note

All claims expressed in this article are solely those of the authors and do not necessarily represent those of their affiliated organizations, or those of the publisher, the editors and the reviewers. Any product that may be evaluated in this article, or claim that may be made by its manufacturer, is not guaranteed or endorsed by the publisher.

## References

[B1] Abs E. Goorden S. M. I. Schreiber J. Overwater I. E. Hoogeveen-Westerveld M. Bruinsma C. F. (2013). TORC1-dependent epilepsy caused by acute biallelicTsc1deletion in adult mice. *Ann. Neurol.* 74 569–579. 10.1002/ana.23943 23720219

[B2] Amir R. E. van den Veyver I. B. Wan M. Tran C. Q. Francke U. Zoghbi H. Y. (1999). Rett syndrome is caused by mutations in X-linked MECP2, encoding methyl-CpG-binding protein 2. *Nat. Genet.* 23 185–188. 10.1038/13810 10508514

[B3] Antoine M. W. Langberg T. Schnepel P. Feldman D. E. (2019). Increased excitation-inhibition ratio stabilizes synapse and circuit excitability in four autism mouse models. *Neuron* 101 648–661.e4. 10.1016/j.neuron.2018.12.026 30679017 PMC6733271

[B4] Atallah B. V. Bruns W. Carandini M. Scanziani M. (2012). Parvalbumin-expressing interneurons linearly transform cortical responses to visual stimuli. *Neuron* 73 159–170. 10.1016/j.neuron.2011.12.013 22243754 PMC3743079

[B5] Banerjee A. Rikhye R. V. Breton-Provencher V. Tang X. Li C. Li K. (2016). Jointly reduced inhibition and excitation underlies circuit-wide changes in cortical processing in Rett syndrome. *Proc. Natl. Acad. Sci. U.S.A.* 113 E7287–E7296. 10.1073/pnas.1615330113 27803317 PMC5135376

[B6] Bateup H. S. Johnson C. A. Denefrio C. L. Saulnier J. L. Kornacker K. Sabatini B. L. (2013). Excitatory/inhibitory synaptic imbalance leads to hippocampal hyperexcitability in mouse models of tuberous sclerosis. *Neuron* 78 510–522. 10.1016/j.neuron.2013.03.017 23664616 PMC3690324

[B7] Blackman M. P. Djukic B. Nelson S. B. Turrigiano G. G. A. (2012). Critical and cell-autonomous role for MeCP2 in synaptic scaling up. *J. Neurosci.* 32 13529–13536. 10.1523/jneurosci.3077-12.2012 23015442 PMC3483036

[B8] Blumenfeld H. (2005). Cellular and network mechanisms of spike-wave seizures. *Epilepsia* 46 21–33. 10.1111/j.1528-1167.2005.00311.x 16302873

[B9] Born H. A. Dao A. T. Levine A. T. Lee W. L. Mehta N. M. Mehra S. (2017). Strain-dependence of the angelman syndrome phenotypes in Ube3a maternal deficiency mice. *Sci. Rep.* 7:8451. 10.1038/s41598-017-08825-x 28814801 PMC5559514

[B10] Brown C. O. Uy J. Murtaza N. Rosa E. Alfonso A. Xing S. (2021). Disruption of the autism-associated gene SCN2A alters synaptic development and neuronal signaling in patient iPSC-glutamatergic neurons. *bioRxiv* [Preprint] 10.1101/2021.09.14.460368 PMC10824931 38293651

[B11] Catterall W. A. (2017). Forty years of sodium channels: structure, function, pharmacology, and epilepsy. *Neurochem. Res.* 42 2495–2504. 10.1007/s11064-017-2314-9 28589518 PMC5693772

[B12] Chao H. T. Chen H. Samaco R. C. Xue M. Chahrour M. Yoo J. (2010). Dysfunction in GABA signalling mediates autism-like stereotypies and Rett syndrome phenotypes. *Nature* 468 263–269. 10.1038/nature09582 21068835 PMC3057962

[B13] Chao H.-T. Zoghbi H. Y. Rosenmund C. (2007). MeCP2 controls excitatory synaptic strength by regulating glutamatergic synapse number. *Neuron* 56 58–65. 10.1016/j.neuron.2007.08.018 17920015 PMC2198899

[B14] Chen R. Z. Akbarian S. Tudor M. Jaenisch R. (2001). Deficiency of methyl-CpG binding protein-2 in CNS neurons results in a Rett-like phenotype in mice. *Nat. Genet.* 27 327–331. 10.1038/85906 11242118

[B15] Dani V. S. Chang Q. Maffei A. Turrigiano G. G. Jaenisch R. Nelson S. B. (2005). Reduced cortical activity due to a shift in the balance between excitation and inhibition in a mouse model of Rett syndrome. *Proc. Natl. Acad. Sci. U.S.A.* 102 12560–12565. 10.1073/pnas.0506071102 16116096 PMC1194957

[B16] De Lombares C. Heude E. Alfama G. Fontaine A. Hassouna R. Vernochet C. (2019). Dlx5 and Dlx6 expression in GABAergic neurons controls behavior, metabolism, healthy aging and lifespan. *Aging* 11 6638–6656. 10.18632/aging.102141 31514171 PMC6756896

[B17] Depaulis A. Charpier S. (2018). Pathophysiology of absence epilepsy: insights from genetic models. *Neurosci. Lett.* 667 53–65. 10.1016/j.neulet.2017.02.035 28216336

[B18] Dong Q. Kim J. Nguyen L. Bu Q. Chang Q. (2020). An astrocytic influence on impaired tonic inhibition in hippocampal CA1 pyramidal neurons in a mouse model of Rett syndrome. *J. Neurosci.* 40 6250–6261. 10.1523/jneurosci.3042-19.2020 32616668 PMC7406277

[B19] Eaton M. Zhang J. Ma Z. Park A. C. Lietzke E. Romero C. M. (2021). Generation and basic characterization of a gene-trap knockout mouse model of Scn2a with a substantial reduction of voltage-gated sodium channel Nav1.2 expression. *Genes Brain Behav.* 20:e12725. 10.1111/gbb.12725 33369088

[B20] Gemelli T. Berton O. Nelson E. D. Perrotti L. I. Jaenisch R. Monteggia L. M. (2006). Postnatal loss of Methyl-CpG binding protein 2 in the forebrain is sufficient to mediate behavioral aspects of Rett syndrome in mice. *Biol. Psychiatry* 59 468–476. 10.1016/j.biopsych.2005.07.025 16199017

[B21] Glaze D. G. Percy A. K. Skinner S. Motil K. J. Neul J. L. Barrish J. O. (2010). Epilepsy and the natural history of Rett syndrome. *Neurology* 74 909–912. 10.1212/wnl.0b013e3181d6b852 20231667 PMC2836870

[B22] Gorski J. A. Talley T. Qiu M. Puelles L. Rubenstein J. L. R. Jones K. R. (2002). Cortical excitatory neurons and glia, but not GABAergic neurons, are produced in the Emx1-expressing lineage. *J. Neurosci.* 22 6309–6314. 10.1523/jneurosci.22-15-06309.2002 12151506 PMC6758181

[B23] Gould E. Kim J. H. (2021). SCN2A contributes to oligodendroglia excitability and development in the mammalian brain. *Cell Rep.* 36:109653. 10.1016/j.celrep.2021.109653 34496232 PMC8486143

[B24] Guy J. Hendrich B. Holmes M. Martin J. E. Bird A. (2001). A mouse Mecp2-null mutation causes neurological symptoms that mimic Rett syndrome. *Nat. Genet.* 27 322–326. 10.1038/85899 11242117

[B25] He H. Shen W. Zheng L. Guo X. Cline H. T. (2018). Excitatory synaptic dysfunction cell-autonomously decreases inhibitory inputs and disrupts structural and functional plasticity. *Nat. Commun.* 9:2893. 10.1038/s41467-018-05125-4 30042473 PMC6057951

[B26] Hippenmeyer S. Vrieseling E. Sigrist M. Portmann T. Laengle C. Ladle D. R. (2005). A developmental switch in the response of DRG neurons to ETS transcription factor signaling. *PLoS Biol.* 3:e159. 10.1371/journal.pbio.0030159 15836427 PMC1084331

[B27] Hirrlinger P. G. Scheller A. Braun C. Hirrlinger J. Kirchhoff F. (2006). Temporal control of gene recombination in astrocytes by transgenic expression of the tamoxifen-inducible DNA recombinase variant CreERT2. *Glia* 54 11–20. 10.1002/glia.20342 16575885

[B28] Horsthemke B. Wagstaff J. (2008). Mechanisms of imprinting of the prader-willi/angelman region. *Am. J. Med. Genet. A* 146A 2041–2052. 10.1002/ajmg.a.32364 18627066

[B29] Huibregtse J. M. Scheffner M. Howley P. M. (1993). Cloning and expression of the cDNA for E6-AP, a protein that mediates the interaction of the human papillomavirus E6 oncoprotein with p53. *Mol. Cell. Biol.* 13 775–784. 10.1128/mcb.13.2.775 8380895 PMC358960

[B30] Idro R. Gwer S. Kahindi M. Gatakaa H. Kazungu T. Ndiritu M. (2008). The incidence, aetiology and outcome of acute seizures in children admitted to a rural Kenyan district hospital. *BMC Pediatr.* 8:5. 10.1186/1471-2431-8-5 18261215 PMC2270816

[B31] Ito-Ishida A. Ure K. Chen H. Swann J. W. Zoghbi H. Y. (2015). Loss of MeCP2 in parvalbumin-and somatostatin-expressing neurons in mice leads to distinct Rett syndrome-like phenotypes. *Neuron* 88 651–658. 10.1016/j.neuron.2015.10.029 26590342 PMC4656196

[B32] Jiang Y. Armstrong D. Albrecht U. Atkins C. M. Noebels J. L. Eichele G. (1998). Mutation of the angelman ubiquitin ligase in mice causes increased cytoplasmic p53 and deficits of contextual learning and long-term potentiation. *Neuron* 21 799–811. 10.1016/s0896-6273(00)80596-6 9808466

[B33] Kepecs A. Fishell G. (2014). Interneuron cell types are fit to function. *Nature* 505 318–326. 10.1038/nature12983 24429630 PMC4349583

[B34] Kishino T. Lalande M. Wagstaff J. (1997). UBE3A/E6-AP mutations cause Angelman syndrome. *Nat. Genet.* 15 70–73. 10.1038/ng0197-70 8988171

[B35] Knopf A. (2020). Autism prevalence increases from 1 in 60 to 1 in 54: CDC. *Brown Univ. Child Adolesc. Psychopharmacol. Update* 22 6–7. 10.1002/cpu.30499

[B36] Koene L. M. Niggl E. Wallaard I. Proietti-Onori M. Rotaru D. C. Elgersma Y. (2021). Identifying the temporal electrophysiological and molecular changes that contribute to TSC-associated epileptogenesis. *JCI Insight* 6:e150120. 10.1172/jci.insight.150120 34877936 PMC8675202

[B37] Li T. Tian C. Scalmani P. Frassoni C. Mantegazza M. Wang Y. (2014). Action potential initiation in neocortical inhibitory interneurons. *PLoS Biology* 12:e1001944. 10.1371/journal.pbio.1001944 25203314 PMC4159120

[B38] Lioy D. T. Garg S. K. Monaghan C. E. Raber J. Foust K. D. Kaspar B. K. (2011). A role for glia in the progression of Rett’s syndrome. *Nature* 475 497–500. 10.1038/nature10214 21716289 PMC3268776

[B39] Luo L. Ambrozkiewicz M. C. Benseler F. Chen C. Dumontier E. Falkner S. (2020). Optimizing nervous system-specific gene targeting with cre driver lines: prevalence of germline recombination and influencing factors. *Neuron* 106 37–65. 32027825 10.1016/j.neuron.2020.01.008 PMC7377387

[B40] Lüttjohann A. van Luijtelaar G. (2015). Dynamics of networks during absence seizure’s on- and offset in rodents and man. *Front. Physiol.* 6:16. 10.3389/fphys.2015.00016 25698972 PMC4318340

[B41] Madisen L. Zwingman T. A. Sunkin S. M. Oh S. W. Zariwala H. A. Gu H. (2010). A robust and high-throughput Cre reporting and characterization system for the whole mouse brain. *Nat. Neurosci.* 13 133–140. 10.1038/nn.2467 20023653 PMC2840225

[B42] Matsuura T. Sutcliffe J. S. Fang P. Galjaard R.-J. Jiang Y. Benton C. S. (1997). De novo truncating mutations in E6-AP ubiquitin-protein ligase gene (UBE3A) in Angelman syndrome. *Nat. Genet.* 15 74–77. 10.1038/ng0197-74 8988172

[B43] McCafferty C. David F. Venzi M. Lõrincz M. L. Delicata F. Atherton Z. (2018). Cortical drive and thalamic feed-forward inhibition control thalamic output synchrony during absence seizures. *Nat. Neurosci.* 21 744–756. 10.1038/s41593-018-0130-4 29662216 PMC6278913

[B44] Meng X. Wang W. Lu H. He L. J. Chen W. Chao E. (2016). Manipulations of MeCP2 in glutamatergic neurons highlight their contributions to Rett and other neurological disorders. *Elife* 5:e14199. 10.7554/eLife.14199 27328325 PMC4946906

[B45] Miyamoto H. Tatsukawa T. Shimohata A. Yamagata T. Suzuki T. Amano K. (2019). Impaired cortico-striatal excitatory transmission triggers epilepsy. *Nat. Commun.* 10:1917. 10.1038/s41467-019-09954-9 31015467 PMC6478892

[B46] Miyazaki H. Oyama F. Inoue R. Aosaki T. Abe T. Kiyonari H. (2014). Singular localization of sodium channel β4 subunit in unmyelinated fibres and its role in the striatum. *Nat. Commun.* 5:5525. 10.1038/ncomms6525 25413837

[B47] Monory K. Massa F. Egertová M. Eder M. Blaudzun H. Westenbroek R. (2006). The endocannabinoid system controls key epileptogenic circuits in the hippocampus. *Neuron* 51 455–466. 10.1016/j.neuron.2006.07.006 16908411 PMC1769341

[B48] Mossner J. Batista-Brito R. Pant R. Cardin J. (2020). Developmental loss of MeCP2 from VIP interneurons impairs cortical function and behavior. *Elife* 9:e55639. 10.7554/elife.55639 32343226 PMC7213975

[B49] Ogiwara I. Miyamoto H. Tatsukawa T. Yamagata T. Nakayama T. Atapour N. (2018). Nav1.2 haplodeficiency in excitatory neurons causes absence-like seizures in mice. *Commun. Biol.* 1:96. 10.1038/s42003-018-0099-2 30175250 PMC6115194

[B50] Operto F. Mazza R. Pastorino G. Verrotti A. Coppola G. (2019). Epilepsy and genetic in Rett syndrome: a review. *Brain Behav.* 9:e01250. 30929312 10.1002/brb3.1250 PMC6520293

[B51] Panayiotopoulos C. P. (2001). Treatment of typical absence seizures and related epileptic syndromes. *Paediatr. Drugs* 3 379–403. 10.2165/00128072-200103050-00006 11393330

[B52] Pinault D. O’Brien T. J. (2005). Cellular and network mechanisms of genetically-determined absence seizures. *Thalamus Relat. Syst.* 3, 181–203. 10.1017/S1472928807000209 21909233 PMC3168114

[B53] Qiu Z. Sylwestrak E. L. Lieberman D. N. Zhang Y. Liu X.-Y. Ghosh A. (2012). The Rett syndrome protein MeCP2 regulates synaptic scaling. *J. Neurosci.* 32 989–994. 10.1523/JNEUROSCI.0175-11.2012 22262897 PMC3711796

[B54] Rubenstein J. L. R. Merzenich M. M. (2003). Model of autism: increased ratio of excitation/inhibition in key neural systems. *Genes Brain Behav.* 2 255–267. 10.1034/j.1601-183x.2003.00037.x 14606691 PMC6748642

[B55] Sanders S. Campbell A. Cottrell J. Moller R. Wagner F. Auldridge A. (2018). Progress in understanding and treating SCN2A-mediated disorders. *Trends Neurosci.* 41 442–456. 10.1016/j.tins.2018.03.011 29691040 PMC6015533

[B56] Satterstrom F. K. Kosmicki J. A. Wang J. Breen M. S. de Rubeis S. An J. Y. (2020). Large-scale exome sequencing study implicates both developmental and functional changes in the neurobiology of autism. *Cell* 180 568–584.e23. 10.1016/j.cell.2019.12.036 31981491 PMC7250485

[B57] Silva-Santos S. van Woerden G. M. Bruinsma C. F. Mientjes E. Jolfaei M. A. Distel B. (2015). Ube3a reinstatement identifies distinct developmental windows in a murine Angelman syndrome model. *J. Clin. Invest.* 125 2069–2076. 10.1172/jci80554 25866966 PMC4463212

[B58] Spratt P. W. Ben-Shalom R. Keeshen C. M. Burke K. J. Clarkson R. L. Sanders S. J. (2019). The autism-associated gene Scn2a contributes to dendritic excitability and synaptic function in the prefrontal cortex. *Neuron* 103 673–685.e5. 10.1016/j.neuron.2019.05.037 31230762 PMC7935470

[B59] Spratt P. W. E. Alexander R. P. D. Ben-Shalom R. Sahagun A. Kyoung H. Keeshen C. M. (2021). Paradoxical hyperexcitability from NaV1.2 sodium channel loss in neocortical pyramidal cells. *Cell Rep.* 36:109483. 10.1016/j.celrep.2021.109483 34348157 PMC8719649

[B60] Taniguchi H. He M. Wu P. Kim S. Paik R. Sugino K. (2011). A resource of Cre driver lines for genetic targeting of GABAergic neurons in cerebral cortex. *Neuron* 71 995–1013. 10.1016/j.neuron.2011.12.010 21943598 PMC3779648

[B61] Terlau J. Yang J. Khastkhodaei Z. Seidenbecher T. Luhmann H. J. Pape H. (2020). Spike-wave discharges in absence epilepsy: segregation of electrographic components reveals distinct pathways of seizure activity. *J. Physiol.* 598 2397–2414. 10.1113/jp279483 32144956

[B62] Ure K. Lu H. Wang W. Ito-Ishida A. Wu Z. He L. (2016). Restoration of Mecp2 expression in GABAergic neurons is sufficient to rescue multiple disease features in a mouse model of Rett syndrome. *Elife* 5:e14198. 10.7554/eLife.14198 27328321 PMC4946897

[B63] Vong L. Ye C. Yang Z. Choi B. Chua S. Lowell B. B. (2011). Leptin action on GABAergic neurons prevents obesity and reduces inhibitory tone to POMC neurons. *Neuron* 71 142–154. 10.1016/j.neuron.2011.05.028 21745644 PMC3134797

[B64] Wallace M. van Woerden G. Elgersma Y. Smith S. Philpot B. (2017). Ube3a loss increases excitability and blunts orientation tuning in the visual cortex of Angelman syndrome model mice. *J. Neurophysiol.* 118 634–646. 10.1152/jn.00618.2016 28468997 PMC5511875

[B65] Wallace M. L. Burette A. C. Weinberg R. J. Philpot B. D. (2012). Maternal loss of Ube3a produces an excitatory/inhibitory imbalance through neuron type specific synaptic defects. *Neuron* 74 793–800. 10.1016/j.neuron.2012.07.013 22681684 PMC3372864

[B66] Williams C. A. (2005). Neurological aspects of the Angelman syndrome. *Brain Dev.* 27 88–94. 10.1016/j.braindev.2003.09.014 15668046

[B67] Wither R. G. Colic S. Bardakjian B. L. Snead O. C. Zhang L. Eubanks J. H. (2018). Electrographic and pharmacological characterization of a progressive epilepsy phenotype in female MeCP2-deficient mice. *Epilepsy Res.* 140 177–183. 10.1016/j.eplepsyres.2018.01.015 29414525

[B68] Wolff M. Johannesen K. M. Hedrich U. B. S. Masnada S. Rubboli G. Gardella E. (2017). Genetic and phenotypic heterogeneity suggest therapeutic implications in SCN2A-related disorders. *Brain* 140 1316–1336. 10.1093/brain/awx054 28379373

[B69] Wood L. Shepherd G. M. (2010). Synaptic circuit abnormalities of motor-frontal layer 2/3 pyramidal neurons in a mutant mouse model of Rett syndrome. *Neurobiol. Dis.* 38 281–287. 10.1016/j.nbd.2010.01.018 20138994 PMC2854239

[B70] Xue M. Atallah B. V. Scanziani M. (2014). Equalizing excitation–inhibition ratios across visual cortical neurons. *Nature* 511 596–600. 10.1038/nature13321 25043046 PMC4117808

[B71] Yamagata T. Ogiwara I. Mazaki E. Yanagawa Y. Yamakawa K. (2017). Nav1.2 is expressed in caudal ganglionic eminence-derived disinhibitory interneurons: mutually exclusive distributions of Nav1.1 and Nav1.2. *Biochem. Biophys. Res. Commun.* 491 1070–1076. 10.1016/j.bbrc.2017.08.013 28784306

[B72] Zhang J. Chen X. Eaton M. Wu J. Ma Z. Lai S. (2021). Severe deficiency of the voltage-gated sodium channel NaV1.2 elevates neuronal excitability in adult mice. *Cell Rep.* 36:109495. 10.1016/j.celrep.2021.109495 34348148 PMC8382316

[B73] Zhang W. Peterson M. Beyer B. Frankel W. N. Zhang Z. (2014). Loss of MeCP2 from forebrain excitatory neurons leads to cortical hyperexcitation and seizures. *J. Neurosci.* 34 2754–2763. 10.1523/jneurosci.4900-12.2014 24523563 PMC3921436

